# Predicted and Experimental Bending Behaviour of Glulam Bonded by RPF Adhesive

**DOI:** 10.3390/ma17020514

**Published:** 2024-01-21

**Authors:** Tomáš Kytka, Miroslav Gašparík, Lukáš Sahula, David Novák, Elham Karami, Sumanta Das, Martin Sviták

**Affiliations:** Department of Wood Processing and Biomaterials, Faculty of Forestry and Wood Sciences, Czech University of Life Sciences Prague, Kamycka 1176, 165 00 Prague-Suchdol, Czech Republic; kytkat@fld.czu.cz (T.K.); gasparik@fld.czu.cz (M.G.); sahulal@fld.czu.cz (L.S.); novakdavid@fld.czu.cz (D.N.); dass@fld.czu.cz (S.D.); svitakm@fld.czu.cz (M.S.)

**Keywords:** glued laminated timber (GLT), combined glulam, modulus of elasticity (MOE), bending strength (MOR), FEM, modelling, beech, alder, spruce

## Abstract

In this study, alder, spruce, and beech woods were used for homogeneous symmetric, inhomogeneous symmetric (combined) and inhomogeneous non-symmetric glued laminated timber (glulam) beams glued with resorcinol phenol formaldehyde (RPF) adhesive. The aim of this paper is to determine and compare the modulus of elasticity of glulam beams using three methods, i.e., analytical calculation, numerical model (FEM) and experimental testing. As an additional characteristic, the bending strength (MOR) of the beams was determined during experimental testing. Analytical calculation was used to calculate the modulus of elasticity (MOE) of glued laminated timber based on the knowledge of the modulus of elasticity of solid wood and to estimate the location of the neutral axis during bending. According to calculations, for symmetrical combinations, the deviation from the real neutral axis does not exceed 5%. In the case of the modulus of elasticity, the deviation is an average of 4.1% from that of the actual measured beams. The numerical model includes finite element modelling, where the deflection of the modelled beams can be calculated with a deviation of up to 10%. The last method was experimental testing of glued beams using four-point bending, in which, among homogeneous beams, beech glulam beams achieved the highest MOE and MOR, while alder glulam beams achieved the lowest. The combination of wood species resulted in an increase in both MOE and MOR compared to homogeneous spruce and alder beams.

## 1. Introduction

Glued laminated timber (glulam) is a construction material consisting of wooden lamellae glued together using an adhesive. The resulting beam is strong and versatile, and can be used in a variety of construction projects. Glulam is often used as an alternative to traditional construction materials such as steel or concrete. It is a sustainable option because it is made from a renewable resource and has a smaller carbon footprint compared to these other materials [1]. Another great advantage of glulam is that lamellae with better mechanical properties are used for production; therefore, by selecting and combining such lamellae, it is possible to obtain higher values of characteristic strengths than can be achieved with solid wood [2].

Commonly used wood species include especially spruce, which is among the most used wood species due to its properties and good workability [3]. However, with advancing global climate change, spruce stands are beginning to show significant climate change stress [4]. In Central Europe, the main factors include drought and the bark beetle calamity, which decimates stands of softwood species on a large scale [5,6]. An alternative to glulam made from softwood can be the use of glued beams from hardwood species. The most known hardwood is beech, which is known for its good mechanical properties, but has somewhat poorer resistance to biotic degradation and experiences a large change in properties when exposed to moisture change [7]. Another hardwood that is increasing in its use in glued laminated timber is oak. This strong but brittle hardwood is also known for its increased content of extractive substances in the wood and specific behaviour at increased humidity [8]. Softwoods, such as poplar or alder, are also of considerable interest [9]. In Europe, these wood species are mostly not applied in industrial use for glued laminated timber, but they are commonly used for the production of plywood or furniture parts [10]. Glued laminated timber beams made with these species revealed a very promising mechanical behaviour [11].

The term glulam has many different meanings. According to ČSN EN 14080 (2013) [12], it is structural material made from lamellae with different strength classes across the cross-section of a beam. Alternatively, combined glulam, often also called hybrid glulam, is made from lamellae of two or more different types of wood. The term hybrid glulam is more commonly used for glulam bonded to a material other than wood. Typical representatives of hybrid glued laminated timber are glulam reinforced by fibre-reinforced polymer (FRP), mostly carbon-fibre, glass-fibre and wood-concrete composite [13,14,15,16].

The utilisation of a combination of wood species in a glued beam is primarily driven by the distinctive mechanical properties inherent in each wood species. Various wood species possess their own unique characteristics. The combination of different species allows for the strategic exploitation of their individual strengths. This composite approach can yield glulam structures with superior overall strength and durability compared to single wood species materials [17]. Incorporating varied wood species also promotes the efficient utilisation of available resources. Regional availability and sustainable sourcing of different species can be capitalised upon, minimising waste and optimising resource utilisation within the glulam production process [18].

Different wood species exhibit differential responses to environmental stimuli such as humidity, temperature fluctuations and mechanical loading, which promote the occurrence of stress in beams. These stresses may cause dimensional changes in the wood, leading to the onset of shear stress, especially within the surface layers of bonded wood [19]. These micro-cracks can turn into macro-cracks, especially in the direction of the longitudinal axis of the beam, due to longer exposure or displacement of individual elements in relation to one another. This phenomenon results in a reduction in the effective area over which the glued wood transmits shear stress, thus reducing the entire cross-section of the beam and reducing strength even under other modes of stress, especially in bending [20].

During bending, the wood is also exposed to normal forces, which are maximized on the upper and lower horizontal surfaces of the beam. These normal forces cause compressive stress (on the side where the forces are applied) and tensile stress in glued wood [21]. Optimising the magnitude of these normal stresses can be achieved by combining wood species in the surface layers of the beam. In particular, the placement of lamellae from a wood species with higher strength, e.g., beech, on the tensile side of the beam results in an increase in bending strength of up to 70% compared to ordinary glulam, e.g., pine [22]. Positioning the less strong lamellae on the compression side of the beam and the stronger wood species on the lamellae subjected to tension can lead to the induction of deformations in the most compressed lamella beyond its elastic limit. This is associated with a ductile behaviour in that lamella [23].

The strength of bonded joints can also be affected by the occurrence of growth defects, high content of extractives, lower wettability, porosity and permeability [24], and especially the occurrence of biotic factors, which can be diagnosed in several ways, for example, the use of X-ray [25]. This work aims to compare the analytical methods via calculation with a model created using FEM. Both models were verified by experimental testing of glued beams with a static bending test.

## 2. Materials and Methods

### 2.1. Analytical Model

The theoretical model, based on which the estimated modulus of elasticity of glued beams was calculated, is based on Timbolmas et al. (2022) [26]. The model presents an analytical model for predicting the resulting modulus of elasticity based on knowledge of the modulus of elasticity in tension and compression for individual lamellae. By adjusting formulas for our species, it is possible to calculate the predicted value for both homogeneous beams and for combined symmetrical or non-symmetrical combinations. Input values from Table 1 were used for the calculation.

From the values of tensile and compressive moduli of elasticity, it is possible to derive a function for calculating the location of the neutral axis. From this value, it is then possible to calculate the estimated modulus of elasticity for the entire beam. This analytical calculation can accurately predict the approximate value of the modulus of elasticity of the glued beams. For a homogeneous beam (i.e., from one type of wood), the following relationships (Equations (1) and (2)) apply [26]:

(1) $$y_{n}=\frac{\sqrt{E_{c}}\cdot h}{\sqrt{E_{c}}+\sqrt{E_{t}}}$$

(2) $$MOE_{global}=\left(\frac{4\cdot E_{c}\cdot E_{t}}{{\left(\sqrt{E_{c}}+\sqrt{E_{t}}\right)}^{2}}\right)$$

where *y_n_* is the position of the neutral axis measured from bottom of beam [mm], *MOE_global_* is the global modulus of elasticity of the whole beam [MPa], *E_c_* is the modulus of elasticity in compression [MPa], *E_t_* is the modulus of elasticity in tensile [MPa], and *h* is the height of beam [mm].

For an inhomogeneous asymmetrical beam (Equations (3) and (4)):

(3) $$y_{n}=\frac{\left(t_{1}\cdot \;\left(\frac{E_{c1}}{E_{t1}}\right)\right)+\left(n_{1}\cdot t_{2}\cdot \left(\frac{E_{c2}}{E_{t2}}\right)\right)\cdot \left(\left(n-1\right)\cdot t\right)}{t_{1}\cdot \;\left(\frac{E_{c1}}{E_{t1}}\right)+n_{1}\cdot t_{2}\cdot \left(\frac{E_{c2}}{E_{t2}}\right)}$$

(4) $$MOE_{global}=\frac{2\cdot \left(E_{t1}\cdot \left(3t_{2}-y_{n}\right)\cdot y_{n}^{2}+E_{c1}\cdot {\left(h-y_{n}\right)}^{2}\cdot \left(2h-3t_{2}+y_{n}\right)\right)}{h^{3}}$$

where *y_n_* is the position of the neutral axis measured from bottom of beam [mm], *t_1_* is the thickness of upper lamellae [mm], *t_2_* is the height of bottom lamellae measured from the bottom of beam [mm], *E_c1_* is the modulus of elasticity in compression of upper lamellae [MPa], *E_c2_* is the modulus of elasticity in compression of bottom lamellae [MPa], *E_t1_* is the modulus of elasticity in tensile of upper lamellae [MPa], *E_t2_* is the modulus of elasticity in tensile of bottom layers [MPa], *h* is the height of the beam [mm], *t_2_* is the thickness of bottom lamellae [mm], *t* is the thickness of the lamella, *n* is the number of lamellae in the beam, *n_1_* is the number of bottom lamellae.

For an inhomogeneous symmetrical beam (Equations (5)–(7)):

(5) $$y_{n}=\frac{E_{c3}\cdot t_{4}-\sqrt{E_{c3}\cdot E_{t3}\cdot t_{4}^{2}-2\cdot E_{c4}\cdot \left(E_{c3}-E_{t3}\right)\cdot \left(h-2t_{2}\right)\cdot t_{2}}}{E_{c3}-E_{t3}}\;$$

(6) $$MOE_{global}=\frac{-2\cdot \left(\left(B\right)+6E_{t4}\cdot t_{2}\cdot \left(h-2y_{n}\right)\cdot \left(t_{2}+y_{n}\right)\right)}{h^{3}}\;$$

where:

(7) $$B=E_{c3}\cdot {\left(h-y_{n}\right)}^{2}\cdot \left(h-3t_{2}-y_{n}\right)+E_{t3}\cdot y_{n}^{2}\cdot \left(-3h+3t_{2}+y_{n}\right)$$

where *y_n_* is the position of neutral axis measured from bottom of beam [mm], *E_c3_* is the modulus of elasticity in compression of inner lamellae [MPa], *E_c4_* is the modulus of elasticity in compression of outer lamellae [MPa], *E_t3_* is the modulus of elasticity in tensile of inner lamellae [MPa], *E_t4_* is the modulus of elasticity in tensile of outer layers [MPa], *h* is the height of the beam [mm], *t_2_* is the thickness of bottom lamellae measured from the bottom of the beam [mm], *t_3_* is the thickness of outer lamellae measured from the bottom of the beam [mm], *t_4_* is the thickness of inner lamellae [mm].

### 2.2. Numerical Model

The presented models were created in COMSOL Multiphysics 6.0 software. This software enables the modelling and description of the behaviour of a hypothetical beam and the influence of the characteristics of individual layers.

**Figure 1:**
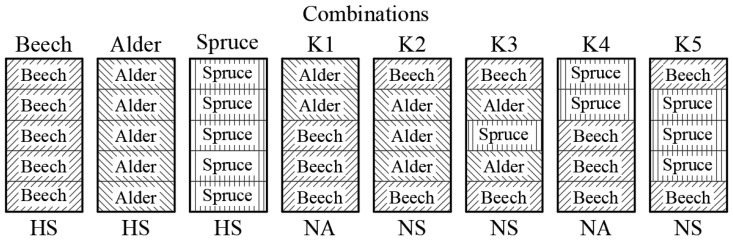
Combination key of glulams. H = Homogenous, S = Symmetrical, N = Non-homogenous, A = Asymmetrical.

A glued beam consisting of 5 layers of lamellae was chosen for this model. The model was divided into three subsections, namely a homogeneous glued beam, where the individual lamellae were always from the same wood species, and an inhomogeneous but symmetrical subsection, where the two outer layers were always made of beech and the central one was made of spruce or alder (K2, K3 and K5), see Figure 1. The last group consists of inhomogeneous asymmetrical beams, where the lower three lamellae are made of beech and the upper two are made of spruce or alder (K1 and K4).

A 3D model was created for the bending test. For this model, the length of the individual lamella was always 1200 mm, the width 60 mm and the height 12 mm. These individual lamellae were connected using the “form assembly” function with a modelled “contact pair”. The “prescribed displacement” function was used to fix the model beam, where u = 0, v = 0 and z = 0 were used for the left support (rotational connection). For the right support, z = 0. A linear brick node was used for the mesh.

Material, here wood, was modelled as an elastic material without considering the failure. Additionally, the model does not consider any wood defects, although there are already studies dealing with this issue [2]. In order to model the elastic region, it is necessary to know several basic material characteristics. These include, in particular, three elastic moduli (*E*_1_, *E*_2_ and *E*_3_), three Poisson ratios (*ν*_1_, *ν*_2_, *ν*_3_) and three shear moduli (*G*_1_, *G*_2_ and *G*_3_).

**Figure 2:**
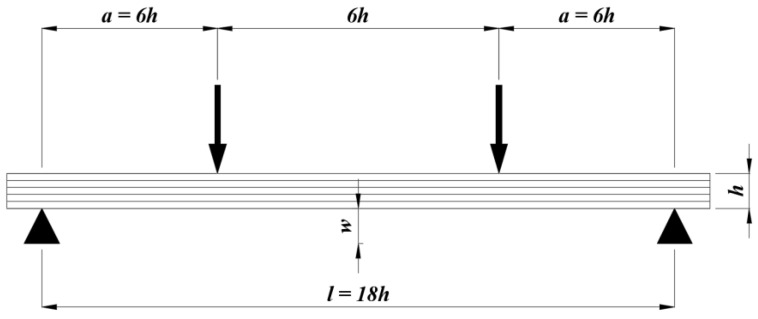
The principle of four-point bending.

The load on the beam was represented by a pair of forces represented by the “edge load” function, symbolising the upper supports of the testing machine. Distances of action of these forces for the arrangement of the static bending test are shown in Figure 2. To determine the load force, the bending strength was determined from the literature. The lowest value was used for load force calculation, so all wood species will fit into the linear region of the stress strain diagram. From the selected wood species, the lowest bending strength occurred for alder. The average bending strength values found were in the range of 44–110 MPa [9], from which were determined the mean value.

From this mean value, the 5% quartile was determined according to ČSN EN 14080 (2013) [12]. As a result, the 5% quantile of the model bending strength of alder was estimated at 64 MPa. From this value, the maximum load force was determined by modifying Equation (8) for bending strength from the standard ČSN EN 408 + A1 (2012) [27].

(8) $$f_{m}=\frac{3\cdot F\cdot a}{b\cdot h^{2}}\to F=\frac{f_{m}\cdot b\cdot h^{2}\;}{3\cdot a}$$

where *f_m_* is the bending strength [N/mm^2^], *F* is the load [N], *a* is the distance between the load point and support [mm], *b* is the width, *h* is the height.

By substituting the variables into the above-mentioned equation, a value of the maximum load force of 12,800 N was obtained. At this value, the bending strength is exceeded, but for the purposes of this article it is necessary to continue only in the linear region, which according to Equation (12) is in the range of 10–40% (see *F_1_ − F_2_*). Therefore, for the maximum loading force of the model, the 40% quantile of this maximum was chosen, which represents 5120 N. All model combinations were loaded with this force and the deflection at the given force was determined; this should reflect the lowest possible loading force, which is still in the linear region of all wood species and combinations used.

The values used in the model also reflect the strength classes determined by ČSN EN 338 (2016) [28]. Based on the counted characteristic, the value for alder wood was used for the numerical model, assigned to strength class C22 (*f_t,0,l,k_* = 13 MPa, *E_t,0,l,mean_* = 10,000 MPa, *ρ_l,k_* = 340 kg/m^3^), which when used in homogeneous glued laminated timber corresponds to the class GL22c (*f_m,g,k_* = 22 MPa). However, there is an assumption that the real bending strength of other types of wood and combinations will be higher, so these modelled deflections will be compared with the real values found during physical testing of the glued samples and recalculated based on the real average load force found during the mechanical test.

The modelled problem is in the domain of linear deformations. The result is therefore the amount of stress that occurs in the beam. Furthermore, displacement is compared with the deflection of real sample (*w_2_*). The modelling of the glued joint is mainly influenced by the properties of the used adhesive and its thickness, and on the basis of the so-called penalty. This expresses the penalty stiffness of the interface and is expressed by Equation (9).

(9) $$K_{i}=\frac{\alpha \cdot E_{l}}{h_{1}}$$

where *K_i_* is the interface stiffness [N/mm^3^], *α* is a parameter much larger than 1, here 50 [29,30], *E_l_* is the longitudinal Young’s modulus of timber [MPa], *h_1_* is the single wood lamella height [mm].

The elastic properties of individual lamellae are not affected by the cohesive layer if E_l_ << Ki∙t. Additionally, too large values of α can cause a large deviation in the calculation of interface stiffness. For values higher than 50, the loss of accuracy is about 2%, which is acceptable accuracy [30].

Stiffness was also calculated. The stiffness was calculated in the linear region (up to 40% of the maximum load) based on Equation (10) [31]:

(10) $$K=\frac{∆F}{∆w}$$

where *K* is the stiffness [N/mm], ∆*F* is the increment of applied load [N], ∆*w* is the corresponding displacement [mm].

### 2.3. Sample Preparation

A total of three wood species were chosen to produce the samples: Norway spruce (*Picea abies* (L.) H. Karst.), alder (*Alnus glutinosa* (L.) Gaertn.) and beech (*Fagus silvatica* L.). Lumber was purchased from a local lumber supplier. The thickness of the lumber was set at 50 mm for yield reasons. The lamellae were scanned using a surface laser scanner and X-ray scanner (WoodEye doubleside, Microtec, Italy), thanks to which defect-free areas were selected in the slats, from which lamellae intended for glulam gluing were later cut.

The individual lamellae were produced in dimensions (l × w × h) of 1300 × 70 × 12 mm. The thickness of 12 mm was achieved by planing on both sides, thereby obtaining sufficiently smooth and flawless surfaces for gluing. A total of 85 lamellae from beech, 60 from alder and 55 from spruce were used. All lamellae were without defects and the angle of the fibres did not deviate by more than 5° from the longitudinal axis. The dimensions and weight of the lamellae were subsequently measured, and were inserted into the formula for calculating the density. The density of the individual lamellae then played a major role in forming the positional key; see Figure 1. The position was determined in such a way that the lamellae with the same or very similar density values were located as close as possible to each other in one wood species. This suppressed the effect of the variation in beam density with height, unless it was desired, as in the case of combinations.

The resorcinol phenol formaldehyde glue PRF system 1711/2520 from Akzo Nobel N.V. (Stockholm, Sweden) was chosen for gluing the beams. This adhesive consists of two components whose properties are listed in Table 2.

For the production of glued laminated wood, the conditions specified by the adhesive manufacturer were observed, i.e., adhesive application of 250 g/m^2^, pressing pressure of 1 MPa and pressing time of 8 h at 20 °C. After this time, the beams were taken out of the press, where they hardened for at least one day. Afterwards, the beams were planed and shortened to the required dimensions of 1200 × 60 × 60 mm (l × w × h). The thickness of the beam was not subsequently adjusted, and the final thickness was ensured by pressing itself.

All prepared samples (40 samples = 8 combinations × 5 pieces/combination) were then placed in a Clime Event 2/2000/40/3 air-conditioning chamber (Weiss Umwelttechnik GmbH, Hamburg, Germany) for four weeks under conditions of 65 ± 5% RH and 20 ± 2 °C to achieve an equilibrium moisture content of 12%. Afterwards, the dimensions and weights of all samples were measured, from which the average density of beams was subsequently calculated and the moisture content was verified at 12%.

### 2.4. Testing Methods

#### 2.4.1. Physical Properties

From the physical properties, dimensions and weight were measured for all samples. Density and moisture content (Equation (11)) were then calculated from these values according to ISO 13061-1 (2014) [32] and ISO 13061-2 (2014) [33], respectively.

(11) $$W=\frac{m_{1}-m_{2}}{m_{2}}\times 100\;$$

where *W* is moisture content [%], *m_1_* is the initial mass of the test sample before drying [g], *m_2_* is the oven-dry mass of the specimen [g].

#### 2.4.2. Static Bending Test

The bending test was performed using a TIRA 2850 S E5 testing machine (TIRA, Schalkau, Germany). The arrangement of the test was carried out according to ČSN EN 408 + A1, (2012) [27] on a 4-point bending principle (Figure 2). The displacement speed of the loading head was set to 5 mm/min. At this speed, the failure was reached in 300 ± 120 s. Direct recording of loading force and deflection was performed via a PC connected to testing machine.

The global modulus of elasticity was chosen for the calculation, especially for greater measurement accuracy and easier application of the extensometer, which rested on the underside of the tested samples. Compared to the local modulus of elasticity, however, it also contains a shear modulus of elasticity [34]. The advantage of this use, however, in the case of asymmetrical combinations, is the fact that their neutral axis does not occur in the middle of the height of the samples, so it would have to be found separately. Equation (12) was used to calculate the modulus of elasticity (MOE).

(12) $$E_{m,g}=\frac{3\;a\;l^{2}-4\;a^{3}}{2\;b\;h^{3}\;\left(2\;\frac{w_{2}-w_{1}}{F_{2}-F_{1}}\right)-\frac{6\;a\;}{5\;G\;b\;h}}$$

where *E_m,g_* is the global modulus of elasticity [N/mm^2^], *a* is the distance between a loading position and nearest support [mm], *l* is the length of the beam between supports [mm], *b* is the width of the sample, *h* is the height of sample, *F_2_ − F_1_* is the increment of the load [N] at 10% and 40% of F_max_, *w_2_ − w_1_* is the increment of displacement corresponding to *F_2_ − F_1_* in [mm], *G* is the shear modulus [MPa].

At the same time, the ultimate bending strength was also determined, which according to the standard ČSN EN 408 + A1 (2012) [27] was calculated using Equation (13):

(13) $$f_{m}=\frac{3\;F\;a}{b\;h^{2}}$$

where *f_m_* is the bending strength [N/mm^2^], *F* is the load [N], *a* is the distance between the load point and support [mm], *b* is the width, *h* is the height.

#### 2.4.3. Statistical Analysis

Tukey’s test was used to calculate the statistical significance of the difference between the measured combinations. An analysis of variance (ANOVA) was used to evaluate the dispersion and plot the graphs. Both methods were implemented using the software Statistica 14 (TIBCO Software Inc., Palo Alto, CA, USA).

## 3. Results and Discussion

### 3.1. Analytical Model

The results of the analytical model are presented in Table 3. For a better comparison of the results, the results of the measured values of the modulus of elasticity from previous measurements are also included [35], where the mechanical properties of the same combinations were compared depending on the temperature change. For comparison, only values with the same wood moisture without the influence of temperature changes were selected.

The first result finds the location of the neutral axis in the glued beam (*y_n_*) measured from the bottom of the beam. In the general theory of elasticity, in homogeneous isotropic materials with the same moduli of elasticity, the neutral axis is located at the centre of the beam. However, wood is an inherently anisotropic and highly nonhomogeneous material. Even the neutral axis of clear wood samples deviates from the hypothetical centroid of the beam [36]. For the purpose of symmetrical combinations, we assume that the neutral axis is located at the centre of the height of the bent beam, which is 30 mm in this case. The difference is insignificant, up to 1.5 mm. For symmetrical inhomogeneous beams, such as combinations of K2 and K5, according to theory, the neutral axis should also be located in the centre of the beam. There is an interesting effect here, where in the case of K2, whose middle layers are alder, there is a slight overestimation, while in the K5 combination, where the middle layers are made of spruce, there is a slight underestimation. In both cases, the difference does not exceed 3 mm, which, with a beam height of 60 mm, constitutes a deviation of up to 5%.

In the case of an inhomogeneous symmetrical beam with combined central lamella (K3), the average of both wood types was used for the calculation values, and the result corresponds to the theory with the same deviation as in the case of K2 and K5. For non-symmetrical beams, such as K1 (beech + alder) and K4 (beech + spruce), there is a noticeable shift in the neutral axis towards the pressure zone. This fact is attributed to the use of woods with a lower modulus of elasticity than beech. In addition, compared to spruce, alder has a lower MOE of about 20%, which causes a deviation in the neutral axis even more towards the pressure zone.

An interesting effect that occurs during bending is the so-called displacement of the neutral axis. Shim et al. (2009) [37] predicted and subsequently experimentally verified the location of the neutral axis and the prediction of the modulus of elasticity based on the Transformed Section Method. They tested a homogenous beam of pine wood glued with resorcinol glue, which had an average modulus of elasticity of 8.6 MPa and an estimated modulus of 11.4 MPa, which is 25% higher than the real value. In the case of a combination of wood species (pine and larch), the predicted MOE was 11.7 MPa and the real result was 10 MPa (14% difference). In the case of the location of the neutral axis, according to the method used by them, the location of the neutral axis differed from the centre of the beam in a homogeneous pine beam by an average of 1.5 mm.

For our homogeneous beams, the average deviation from the centre of the beam is 0.9 mm. In the case of combined symmetrical glulam, Shim et al. (2009) [37] found that the course of the neutral axis deviates by an average of 0.9 mm from the real measurement, which in our case is 1.2 mm (K2, K3 and K5). Therefore, despite the use of a different method, it is possible to see an analogy to our beams from their results. The predicted modulus of elasticity is higher than the real value. Additionally, homogeneous beams show higher deviations from real values than combinations of wood species. The exception in this case is the combination K5, which, when tested using RPF adhesive, showed higher strength than a homogeneous beech beam.

Alternatively, Rescalvo et al. (2020) [31], using the Parallel Axes Theorem, estimated the modulus of elasticity for glued homogeneous and combined poplar and pine wood. Their findings show that the calculated modulus of elasticity for homogeneous poplar glulam of strength class GL20h (lamellae T10) is 7% lower than what they measured on real samples. In our case, the lowest strength class is alder, GL22h (lamellae T12), which, however, shows a +9% higher difference compared to the measured values. Similarly, for the second wood species, pine (GL32h—lamellae T24), they found a difference of −6.6% lower compared to the measured value. Analogously, in our case the spruce (GL32h—lamellae T26) shows slightly lower values (difference—2.3%) than the measured values. These differences clearly indicate a more homogeneous structure of softwoods, while hardwoods, especially less dense ones, show greater variability, and therefore greater difficulty in finding an accurate model.

However, an interesting effect occurs with a combination of wood species. Rescalvo et al. (2020) [31] used a combination of softwood and hardwood wood (pine and poplar), where softwood was in the outer layers. In our case, two symmetrical inhomogeneous combinations were used (K2—beech and alder, K5—beech and spruce), where in both cases the outer lamellae were made of beech. In the K5 combination, in our case, the difference between predicted and measured value was −14.4%, while Rescalvo et al. (2020) [31] presented a decrease of −9.9%, which is a noticeable difference, but compared with our estimation results for PUR, the difference is only −8.3%, which corresponds to their results. The difference occurs in combination K2, which is entirely made up of hardwood species, when the difference reaches +4.9%. It can be seen from this that the method used by us is very accurate for combinations of hardwood and softwood species, but shows an overestimation of the result for combinations of two hardwood species.

The third method is that of improved constant section glulam. This was used by Cheng and Hu (2011) [38], who tested poplar glulams on which they estimated the location of the neutral axis and the modulus of elasticity of a glued homogeneous symmetrical beam. Their result is that in the case of unreinforced beams glued with methylene diphenyl diisocyanate (MDI), the location of the neutral axis differs from the axis of the beam by an average of 0.9%, and maximally 4%. This difference corresponds with our results, where the largest differences in homogeneous combinations reach 1.4%. Additionally, their estimate of the modulus of elasticity shows a slight deviation from the measured value, on average by 1.7%. Compared to RPF, our results show larger deviations on average, of 5.6% (max. 9%); however, compared to previous research by Kytka et al. (2022) [35], in which glulam samples were glued with PUR glue, this average deviation is lower (3.7%).

### 3.2. Numerical Model

The result of the numerical model is a model that describes the behaviour of glued laminated timber. The result of the numerical model is shown in Table 4.

**Figure 3:**
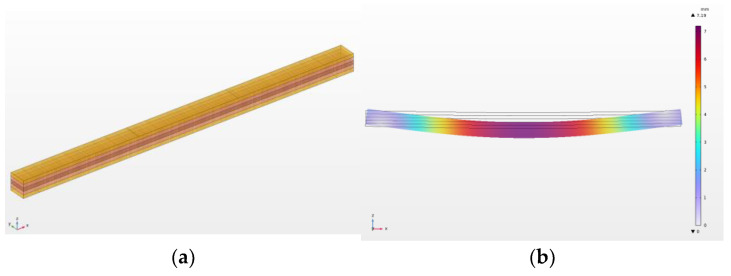
Beam model: (**a**) combination K3, (**b**) deflection of combination K3.

First, the value for all combinations was estimated at a load force of 5120 N (F_m_). The table shows that the highest deflection occurs in alder (10.7 mm), while the lowest is in beech (6.7 mm). Spruce is located approximately in the middle of this interval. Combinations of wood species show very similar characteristics. Combinations in which alder is represented (K1, K2 and K3) show a significant reduction in deflection when alder wood is combined with beech. Differences occur in the arrangement of individual lamellae in the examined combinations. Here, it is fundamentally confirmed that the symmetrical arrangement (K2 and K3—beech in the outer lamellae, alder in the inner ones (see Figure 3)) basically has better characteristics than the asymmetrical arrangement (K1). This effect can be explained by the action of internal forces in the loaded beam, where higher compressive and tensile stresses occur in outer lamellae. Placing wood with higher moduli of elasticity in this region significantly improves the stiffness of the beam.

Conversely, in the case of the asymmetric combination K1, applying the same load leads to earlier formation of elastic deformations, particularly in the compression zone of the glued beam. In the combinations that contain spruce (K3, K4 and K5), similarly as with alder, deflection is reduced due to the presence of beech lamellae. Spruce wood, however, has a higher modulus of elasticity compared to alder, so it does not differentiate in material properties as for beech × alder. Additionally, the compressive strength of spruce can reach the lower limit of the values measured for beech. For these reasons, there was a reduction in deflection, although the differences were not so distinctive.

The second part of the results represent loading modelling using real load values (*F_2_*) and corresponding deflection values (*w_p2_*), which are described in more detail in the section that presents empirically determined results. It can be observed that combinations of wood species that show a similar load force also have a similar amount of deflection in real testing. In contrast, the modelled variants show a similar trend, but a greater dispersion of values. Combination K2 shows the lowest deflection in both cases, and combination K1 is the highest. For homogeneous beams, the comparison is more complicated. Alder has the lowest load capacity, which is only half that of beech. Even so, the deflection value reaches two-thirds that of the beech values. If the alder were to be loaded with the same load force as the beech, then beams would be in the area of plastic deformations or they would exceed the ultimate bending strength limit. The spruce, in comparison with alder, was loaded with a higher force, but achieved a smaller deflection than the alder.

An important insight is provided by comparing real values (*w_2_*) and model values (*w_p2_*). It can be seen from the table that all modelled combinations show a higher deflection than was measured on real beams. The average deviation from testing is 1.1 mm, which corresponds to an average of 9.6%. In this case, if the model shows a higher deflection than the real beam, then the model is “stricter”, and shows a worse variant than the real value of the deflection and overestimates the whole result.

The last part of the results of the numerical model consists of the calculation of the stiffness of individual combinations. The results show that the beech beam achieves the highest stiffness of the modelled beams, while the alder beam has the lowest stiffness and the spruce beam is approximately the average of both. Combinations with alder show a considerable increase over the homogenous alder beam, more for symmetrical combinations than for asymmetric ones. The situation is different for the physically measured beams, especially because the K5 combination achieves the highest stiffness. However, all the stiffness values calculated from the modelled values show significant underestimation (by 9.6% on average).

An interesting result was found by Melzerová et al. (2014) [39], who modelled the deflection of a glued spruce beam. Their model was enriched by the Latin Hypercube Sampling (LHS) method, which allows the generation of near-random parameters of the sample. The result of their model, as verified by experimental measurement, is a difference of +5.5% overestimation by the model compared to the real measured beam.

In contrast, Rescalvo et al. (2020) [31], who numerically modelled glued beams of pine, poplar and their combination, reached the opposite conclusion. Although they used different types of wood, their results can be compared thanks to their inclusion in strength classes. The stiffness of the pine beams (T24) is close to that of our spruce beams (T26), with the difference between their model being 10% on average, while it is 8.2% for our model. It is similar for other wood species, where their model with poplar (T10) shows a change of 2.4%, while our alder (T13) shows a difference of 8.8%. Additionally, their combination (T30+T8) shows a difference of 2.2%, while our combination approaching the parameters of their combination is K2 (T30 + T13) shows a difference of 10.4%. The average difference is then 4.9% and 9.1% in our case. These differences are due to the different compositions of wood species, which have different properties, and also to their arrangement in the glued beam. However, it can be stated that the deflections and stiffnesses of the proposed beams can be modelled with sufficient accuracy.

### 3.3. Experimental Testing

This part of the results relates to the testing of real beams that were subjected to a static bending test. The modulus of elasticity and bending strength were calculated. For comparison with PUR adhesive, the results from previous research [33] were also used.

#### 3.3.1. Modulus of Elasticity (MOE)

**Figure 4:**
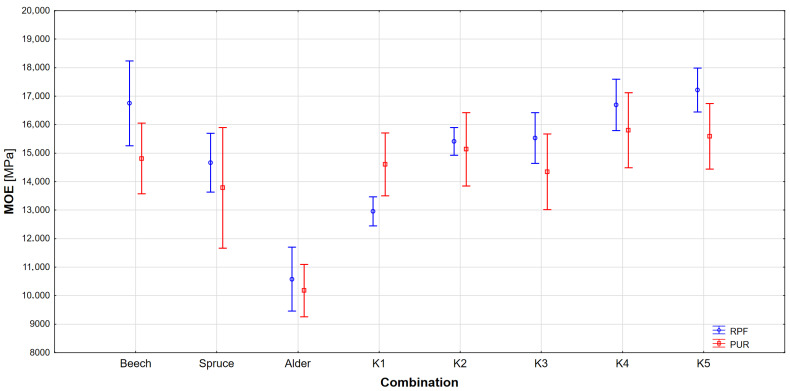
Modulus of elasticity of glulams bonded by RPF and PUR adhesives.

The results of measuring the modulus of elasticity are shown in Figure 4 and Table 5. Results of ANOVA are listed in Table 6, and Tukey’s test was also performed to determine statistical significance (Table 7).

The results show that there is a statistically significant difference among beech, alder and spruce, but Tukey´s test does not show a significant difference among beech and all combinations. When comparing spruce with combinations, there is no significant difference only in combinations K2 and K3, but combinations containing spruce (K4 and K5) show an increased modulus of elasticity compared to spruce. The differences between the combinations themselves can be divided into two categories. Combinations containing spruce have similar results and there is no statistically significant difference between them, but combinations containing alder show a difference, especially in combination K1, which has the lowest modulus of elasticity of all nonhomogeneous combinations. Like for the behaviour of the K1 combination described in the numerical model, the anatomical structure of the alder and the mechanical properties derived from it also make a significant contribution here. These are the lowest of all combinations. Therefore, during compressive stress in the upper part of the glued beam, the overall stiffness and thus the modulus of elasticity of this combination decreases.

When comparing glulams with RPF and PUR adhesives, a trend can be observed in which RPF adhesive achieves higher MOE than PUR adhesive. At the same time, PUR shows a higher dispersion of values. Based on results in Table 6 and Table 7, the combination of wood is shown to be significant factor. Similarly, adhesive is also statistically significant factor affecting the modulus of elasticity of glued laminated timber.

An interesting result was also achieved by Ehrhart et al. (2020) [40], who tested the modulus of elasticity on beech beams glued with PUR glue of different strength classes and their combinations. Their results show that in the case of homogeneous beams (of the same strength class), there is an increase in the modulus of elasticity among classes GL40h, GL48h and GL 55h by 7.8%, and 14.5%, respectively. Comparing our homogeneous softwood combinations of beech (GL32h) and alder (GL22h), there was a difference of 36% between the two classes. A difference also occurs in their combinations formed by beech, but with different strength classes of the inner lamellae, when for the outer lamellae they used a strength class of 11, which was 20 degrees better than in the central lamellae. The result is an increase in the modulus of elasticity by 1.4 and 1.3%, respectively.

The same result was achieved by Šuhajdová et al. (2023) [41], who measured the modulus of elasticity of combined beech and poplar glulam glued with PUR glue. It is evident from their work that when poplar (strength class D18) is used in the central part of the beam and beech (D40) is used in the outer layers, the modulus of elasticity decreases by −0.8% compared to a homogeneous beech beam. On the other hand, by comparing our non-homogeneous beams, when the outer beech layers are 17 (K2), compared to 4 (K5), higher strength classes show differences. In this case, the difference between K2 and homogeneous beech is a decrease of 8.7%, and that in K5 vs. beech is an increase of 2.7%. However, it is clear that in our case we are dealing with different types of wood, which behave differently when subjected to mechanical stress than a beam made of one type of wood. Nevertheless, in our case, there is a noticeable difference in the use of different types of wood, and thus in other strength classes in the central lamellae of the glued beams.

Just as Europe is looking for possibilities of combinations of wood species with spruce and beech, the rest of the world is also looking for alternatives. For example, Aratake et al. (2011) [42] tested Japanese cedar (*Cryptomeria japonica* D. Don) and combined Japanese cedar and Douglas fir (*Pseudotsuga menziesii* Franco) glulam beams. The combined symmetrical beams from both species result in an average increase in the modulus of elasticity of 34.9% compared to the homogeneous Japanese cedar beam. Such a large increase corresponds to the increase in the modulus of elasticity for our combination of alder and beech (K2), where compared to alder there was an increase in the modulus of elasticity by 45.7%.

When comparing symmetrical combinations, Ngadianto et al. (2023) [43] found the same trend as in our case. Their glued beams from tropical woods (*A. mangium, M. eminii, M. azedarach*) glued in a similar configuration to ours (K2 and K5) showed an increase in modulus of elasticity by adding stronger wood to the outer slats, of 17.2% and 21.6%, respectively, while in our case the increase reached 45.7% in combination K2 and 46.8% in K3 compared to glued alder wood. Similarly, combination K5 increased 17.4% compared to glued spruce wood. From these results, it is evident that the increase in the value of the modulus of elasticity is not only due to the arrangement of the lamellae in the beam, but also to the type of wood used, while the larger the difference between the outer and inner layers, the more noticeable the difference.

#### 3.3.2. Bending Strength (MOR)

**Figure 5:**
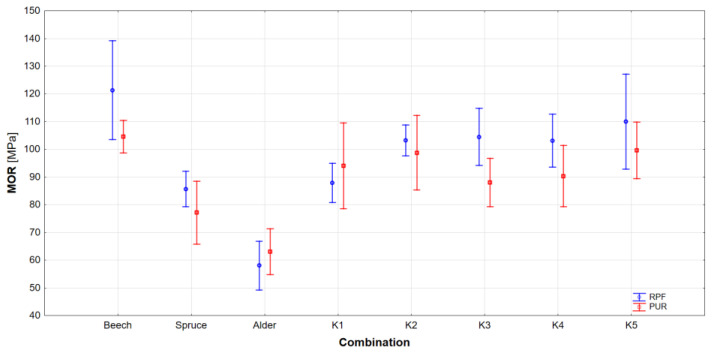
Bending strength of glulams bonded by RPF and PUR adhesives.

The results of the bending strength measurements are shown in Figure 5 and in Table 8. Results of ANOVA are listed in Table 9, and Tukey’s test was also performed to determine statistical significance (Table 10).

It is evident from the results that there is a statistically significant difference between homogeneous beams, with beech beams showing the highest bending strength and alder beams the lowest. Additionally, the comparison of homogeneous and combined beams shows a significant difference in many cases. By comparing the combinations, it can be ascertained that there is no statistically significant difference between them, except for combination K1, which, as in the case of the modulus of elasticity, shows the lowest bending strength of all combinations.

By comparing the adhesives, an interesting observation can be found, namely that higher dispersions are achieved in the case of RPF adhesive. However, overall, the bending strength of beams glued with RPF is higher than in the case of beams glued with PUR adhesive. This effect may be because most adhesives are primarily intended for gluing softwood, which can cause problems in the case of gluing hardwoods, especially due to the content of extractives in the wood, especially tannins. This is also confirmed by Purba et al. (2022) [44], who glued combined glulams from oak and poplar using MUF and PUR adhesive, when the shear strength of homogenous oak beams reached a higher strength, but at the same time a much higher dispersion, than the combination of both species.

Additionally, Tran et al. (2016) [45] tested glued beams from oak and beech glued with MUF adhesive, and examined the influence of finger joints and the number of lamellae used. They found that in the case of homogeneous oak beams, the bending strength is 14% lower than in the case of homogeneous beech beams. Here, again, the influence of the wood species, its anatomical structure and mechanical properties is confirmed.

In the case of combinations of wood species, the cross-section of the beam also plays a significant role, as found by Balász et al. (2020) [46], who combined softwood and hardwood (strength classes C16 and D30). They found that with an increasing cross-section of the beam, there is a higher increase in bending resistance and bending stiffness of the beam in the case of a combined beam with outer lamellae from stronger wood than if a homogeneous beam is used. However, as the cross-section increases, the benefit of this combination diminishes and is highest in the case of small cross-sections.

Rescalvo et al. (2020) [31] measured the flexural strength with a combination of poplar and pine, and when using combinations with strength classes T8 (poplar) and T24 (pine), there was an increase in bending strength by 41.1% compared to homogenous poplar glulam. Similarly, when the strength class of pine was in combination and increased to T30, the bending strength increased by 29.5%.

Ndong Bidzo et al. (2022) [47] tested glued wood from Niove (*Staustia kamernesis*, Ni) and Ozigo (*Dacryodes buettneri*, Oz). In the case of non-symmetric combinations (duo), there was an increase in bending strength compared to the less strong wood (Ozigo) by 9% when Niove was used in the bottom lamella. Furthermore, in the case of symmetrical combinations (trio) there was an increase of 35.4% when using Niove in the outer lamellae. As in the case of these tropical woods, the flexural strength also increases with commonly used woods. In the case of our combinations, the increase is more noticeable in symmetrical combinations (43.8% in K2 vs. alder and 22.1% in K5 vs. spruce). For asymmetric combinations, the increase is lower, but still noticeable (34% in K1 vs. alder and 16.9% in K5 vs. spruce).

## 4. Conclusions

The analytical model reveals a deviation from the neutral axis of up to 5% in symmetrical beams. However, when alder wood is part of the combination, the result tends to be overestimated, whereas with spruce wood, it tends to be underestimated compared to the assumed neutral axis position at the centre of the beam.

In the case of non-symmetrical combinations, the effect of the wood species in the pressure zone is noticeable. When using alder, the deviation from the neutral axis of the beam is higher than when using spruce. The findings show that it depends on the chosen method of estimating the modulus of elasticity and on the wood species used. When calculated using different methods, the deviation is up to 5%. In our case of the modulus of elasticity, the calculated deviation is on average 4.14%, where the lowest is shown by the spruce beam (2.3%) and the highest by the combination K5 (beech + spruce), i.e., 14.38%. However, this deviation is a comparison against measured values.

From the results of the numerical model, it can be seen that the use of FEM modelling can be used for glued laminated timber with an average model accuracy of 9.6%, while the lowest deviation was found for the homogeneous spruce beam model (0.74 mm = 8.16%) and the highest for the K5 combination, with a difference of 1.56 mm = 13.37%. However, such a large difference is compared to experimental values. If the model shows a higher deflection than the real beam, then the model is “tighter” and shows a worse variant than the real deflection value and overestimates the whole result. The properties of the adhesive used also have an influence on the result, which affects the behaviour of the glued joint. Modelling the properties of glued laminated timber is gaining importance, especially due to the acceptable deviation from real values.

The results of experimental testing show a fundamental increase in the modulus of elasticity in the case of a combination of wood species, while this change is considerably influenced by the bending strength of the wood species in the outer layers. From the homogeneous beams, beech had the highest MOE (16,751 MPa) and MOR (121.3 MPa), and alder the lowest (MOE 10,579 MPa, MOR 58 MPa). The combination of wood species increased both MOE and MOR, with the best combination being K5 (MOE 17,216 MPa, MOR 99.6 MPa). In the case of combinations with spruce vs. homogenous spruce, there was an average increase in MOE (15.6%) and MOR (24.4%), and similarly for alder combinations vs. alder by 34% for MOE and 64.8% for MOR. The bending strength of combined glued timber also depends on the wood species used, while the type of wood in the inner layers of the beam does not significantly affect the result. The differences in symmetrical combinations between those with inner layers made of alder or spruce were not statistically significant. The influence of the adhesive, based on ANOVA results, is also evident, i.e., beams glued with RPF adhesive show a higher modulus of elasticity and bending strength.

## Figures and Tables

**Table 1:** Young’s moduli of elasticity for used wood species.

Young´s Modulus		Beech	Alder	Spruce
In compression	[MPa]	14,500	10,000	13,500
In tension	[MPa]	16,000	12,000	14,500

**Table 2:** Selected properties of PRF adhesive from product list.

	1711	2520
Product	PRF adhesive	PRF hardener
Viscosity	3000–8000 mPas	5000–13,000 mPas
Density	Appr. 1150 kg/m^3^	Appr. 1200 kg/m^3^
pH (at time of production)	7.0–9.0 (at 25 °C/77 °F)	3.5–6.0 (at 25 °C/77 °F)
Mixing Ratio (by weight)	100:15, adhesive: hardener	
Glue Spread	170–450 g/m^2^ for laminated beams 250–450 g/m^2^	
Moisture content of wood	8–15% for laminated beams preferably 10–12%	
Pressing Time at20 °C/65% RH, Beech.	0.3 mm glue line thickness	7.5 h
Pressure	Minimum 0.5 MPa for softwood. Minimum 1.0 MPa for hardwood. In laminated beam production: Minimum 0.7 MPa for 33 mm lamellae. Minimum 0.9 MPa for 45 mm lamellae.	
Post curing	1 day at 20 °C/68 °F	

**Table 3:** Analytical model of the position of neutral axis (y_n_) from bottom of the beam and MOE.

			Beech	Alder	Spruce	K1	K2	K3	K4	K5
*y_n_*	[mm]		30.7	31.4	30.5	40	32.5	31.1	39.3	29.9
RPF			Beech	Alder	Spruce	K1	K2	K3	K4	K5
predicted MOE		[GPa]	15.2	10.9	14	11.4	15.7	14.2	14.2	14.4
measured MOE		[GPa]	16.1	10	14.3	12.7	15	14.8	15.6	16.5
Difference		[%]	−5.6	9	−2.3	−10.8	4.9	−4.3	−9.6	−14.4
PUR [36]			Beech	Alder	Spruce	K1	K2	K3	K4	K5
predicted MOE		[GPa]	15.1	10.7	14.3	13.4	15.5	15.1	14.7	15.1
measured MOE		[GPa]	14.8	10.2	13.8	14.6	15.2	14.3	15.8	15.6
Difference		[%]	2.7	6.9	1.5	−27.9	3.5	−1.1	−11.3	−8.3

**Table 4:** Numerical model results.

Property	Measured Values		Predicted Value	Difference (w_p2_-w_2_)		F_m_ (5120 N)	Stiffness K	
	F_2_	w_2_	w_p2_ at F_2_			w_pm_ at F_m_	Real Values	Predicted
	[kN]	[mm]	[mm]	[mm]	[%]	[mm]	[N/mm]	[N/mm]
Beech	10.2	12.2	13.3	1.1	8.3	6.7	838.7	769.6
Spruce	6.2	8.3	9.1	0.7	8.2	7.4	750.5	689.3
Alder	4.9	9.4	10.3	0.9	8.9	10.7	526.3	479.8
K1	7.4	11.2	12.3	1.1	8.8	8.5	661.3	603.1
K2	7.7	9.7	10.8	1.1	10.4	7.2	794.7	711.7
K3	8.5	11	12.0	1	8.6	7.2	778.1	711.1
K4	8.2	10.1	11.3	1.2	10.4	7	812.8	728.6
K5	8.8	10.2	11.7	1.6	13.4	6.8	869.1	752.9
Mean	7.7	10.3	11.3	1.1	9.6	7.7	754	680.8

F_2_ is 40% of F_max_ [N], w_2_ is the displacement corresponding to F_2_ [mm], w_p2_ is the predicted (modelled) value of deflection loaded by F_2_ [mm], F_m_ is the loading force for w_p2_, (5120 N for all combinations) [N], w_pm_ is the predicted (modelled) value of deflection loaded by F_m_ [mm].

**Table 5:** Modulus of elasticity.

MOE [GPa]		Beech			Alder			Spruce			K1		
Combination		Mean	SD	CoV	Mean	SD	CoV	Mean	SD	CoV	Mean	SD	CoV
Adhesive	RPF	16.8	1.1	6.4	10.6	1.1	10.6	14.7	0.7	5	13	0.4	2.8
	PUR	14.8	0.9	6	10.2	0.1	6.5	13.8	1.5	11	14.6	0.8	5.5
Combination		K2			K3			K4			K5		
		Mean	SD	CoV	Mean	SD	CoV	Mean	SD	CoV	Mean	SD	CoV
Adhesive	RPF	15.4	0.4	2.3	15.5	0.6	4.1	16.7	0.7	3.9	17.2	0.6	3.2
	PUR	15.1	0.9	6.1	14.3	1	6.7	15.8	1	6	15.6	0.8	5.3

SD is the standard deviation [GPa], CoV is the coefficient of variation [%].

**Table 6:** ANOVA results for MOE.

	SS	DF	MS	F	*p*
Intercept	1.71193 × 10^10^	1	1.711937 × 10^10^	20,599.67	0.000000 *
Combination	2.65553 × 10^8^	7	3.793622 × 10^7^	45.65	0.000000 *
Adhesive	9.61880 × 10^6^	1	9.618807 × 10^6^	11.57	0.001157 *
Combination × Adhesive	2.12292 × 10^7^	7	3.032748 × 10^6^	3.65	0.002253 *
Error	5.31872 × 10^7^	64	8.310508 × 10^5^		

SS is sum of squares, DF is degrees of freedom, MS is mean of squares, * is statistically significant.

**Table 7:** Tukey’s test for MOE.

Tukey’s Test; MOE [MPa]; RPF Adhesive; *p* < 0.05; Mean Squares = 4836 × 10^2^; Degrees of Freedom = 32.000													
	Beech		Spruce		Alder		K1		K2		K3		K4
Spruce	0.0011	*											
Alder	0.0001	*	0.0001	*									
K1	0.0001	*	0.0104	*	0.0003	*							
K2	0.0771		0.6878		0.0001	*	0.0002	*					
K3	0.1381		0.5133		0.0001	*	0.0002	*	1.0000				
K4	1.0000		0.0015	*	0.0001	*	0.0001	*	0.1032		0.1797		
K5	0.9611		0.0002	*	0.0001	*	0.0001	*	0.0057	*	0.0117	*	0.9285

Where * is statistically significant.

**Table 8:** Bending strength.

MOR [MPa]		Beech			Alder			Spruce			K1		
Combination		Mean	SD	CoV	Mean	SD	CoV	Mean	SD	CoV	Mean	SD	CoV
Adhesive	RPF	121.3	12.9	10.6	58	6.7	11	85.7	4.6	5.3	87.9	5.1	5.8
	PUR	104.6	4.3	4.1	63	5.9	9.4	77.2	8.2	10.6	94.1	11.2	11.9
Combination		K2			K3			K4			K5		
		Mean	SD	CoV	Mean	SD	CoV	Mean	SD	CoV	Mean	SD	CoV
Adhesive	RPF	103.2	4.1	3.9	104.5	7.5	7.1	103.1	6.9	6.7	110	12.4	11.3
	PUR	98.8	9.7	9.8	88	6.3	7.1	90.3	8	8.8	99.6	7.3	7.4

SD is the standard deviation [GPa], CoV is the coefficient of variation [%].

**Table 9:** ANOVA results for MOR.

	SS	DF	MS	F	*p*
Intercept	692,955.5	1	692,955.5	8677.583	0.000000 *
Combination	18,191.6	7	2598.8	32.544	0.000000 *
Adhesive	1057.8	1	1057.8	13.247	0.000547 *
Combination × Adhesive	1393.9	7	199.1	2.494	0.024944 *
Error	5110.8	64	79.9		

SS is sum of squares, DF is degrees of freedom, MS is mean of squares and * is statistically significant.

**Table 10:** Tukey’s test for MOR.

Tukey’s Test; MOR [MPa]; RPF Adhesive; *p* < 0.05; Mean Squares = 82.338; Degrees of Freedom = 32.000													
	Beech		Spruce		Alder		K1		K2		K3		K4
Spruce	0.0001	*											
Alder	0.0001	*	0.0009	*									
K1	0.0002	*	0.9999		0.0004	*							
K2	0.0613		0.0753		0.0001	*	0.1661						
K3	0.0991		0.0458	*	0.0001	*	0.1069		1.0000				
K4	0.0589		0.0783		0.0001	*	0.1718		1.0000		1.0000		
K5	0.5125		0.0041	*	0.0001	*	0.0110	*	0.9334		0.9774		0.9283

Where * is statistically significant.

## Data Availability

Data available on personal request to correspondence author.
